# Postoperative Stomach Volvulus and Pancreatitis Following a Sleeve Gastrectomy

**DOI:** 10.7759/cureus.51118

**Published:** 2023-12-26

**Authors:** Shane B Devine, Sam J King, Bradley J Chatterton, Kurt D Knepley

**Affiliations:** 1 Internal Medicine, Philadelphia College of Osteopathic Medicine, Philadelphia, USA

**Keywords:** bariatric surgery complications, gastric outlet obstruction, dumping syndrome, acute pancreatitis, sleeve gastrectomy

## Abstract

The laparoscopic sleeve gastrectomy is the most common bariatric surgery performed to promote weight loss and improve obesity-related comorbidities. As the number of patients undergoing sleeve gastrectomy increases, so does the prevalence of complications. It is crucial to recognize both common and unusual complications of sleeve gastrectomy to properly diagnose and manage them. We present a unique case of gastric outlet obstruction not visualized on initial imaging and acute pancreatitis following a sleeve gastrectomy. We recommend performing an endoscopy and ordering serum lipase levels in a patient with negative CT scans but persistent postoperative nausea, vomiting, and abdominal pain. The management of postoperative gastric outlet obstruction includes supportive care, balloon dilation of the stenotic area, or gastric bypass if symptoms persist.

## Introduction

The laparoscopic vertical sleeve gastrectomy (SG) was first described in 1988 and has since become the most common bariatric procedure performed globally. According to the National Institutes of Health (NIH) recommendations in 2020, patients with a body mass index (BMI) of 40 kg/m^2^ or more are candidates for bariatric surgery. Additional candidates include patients with either a BMI of 35-39.9 kg/m^2^ with obesity-related comorbidity or a BMI of 30-34.9 kg/m^2^ with uncontrolled type 2 diabetes mellitus (DM2) despite lifestyle changes and exercise [1].

Postoperative complications of SG include worsening or new-onset gastroesophageal reflux disease (GERD), leakage of the staple line, intraluminal and intraabdominal bleeding, and porto-mesenteric vein thrombosis. Worsening or new-onset GERD has been a reported complication in 19% and 23% of patients, respectively [2]. Overall leak rate of the staple line has been reported at 1.5% [3]. Rare complications include intraluminal and intraabdominal bleeding and porto-mesenteric vein thrombosis [4,5]. Additionally, gastric obstruction has been reported as a complication in 0.2-4% of cases. Most obstructions are thought to be due to one of two mechanisms. The first includes mechanical narrowing, most often occurring at the incisura angularis. The second includes maladaptive rotation and twisting of the sleeve caused by improper alignment of the staples along the anterior and posterior gastric walls [6].

Data regarding the complication rate of SG in class 1 obesity (BMI 30-34.9 kg/m^2^) and non-obese patients have recently emerged and suggested the relative safety of the procedure. A retrospective study of 1,300 patients with DM2 and a BMI between 25 and 35 kg/m^2^ who underwent bariatric surgery demonstrated that the rate of individual major complications, including surgical site infection, deep vein thrombosis, pulmonary embolism, stroke, myocardial infarction, pneumonia, acute renal failure, sepsis, septic shock, unplanned intubation, prolonged ventilation (> 48 hr), cardiac arrest, prolonged hospital stay (> 7 days), and mortality was ≤ 0.5% with the exception of postoperative bleeding requiring transfusion or reoperation which occurred in 1.7% and 1.6% of participants, respectively [7].

We present the case of a patient who presented with gastric outlet obstruction not seen on imaging and acute pancreatitis that was initially misdiagnosed as dumping syndrome two weeks after undergoing circumvention tourism SG.

## Case presentation

A 24-year-old female with a past medical history of asthma and pancreatitis presented to the emergency department with complaints of severe abdominal pain, nausea, and vomiting for two weeks duration. Approximately two weeks prior, the patient underwent an elective sleeve gastrectomy procedure in Mexico, as she did not meet the criteria for it to be covered by her insurance in the United States. Immediately following the procedure, the patient experienced severe abdominal pain, persistent nausea and vomiting, and decreased oral intake, resulting in approximately 20-pound weight loss. Abdominal pain was generalized and persistent since the procedure. Emesis was brown and non-bloody. The patient noted having only one bowel movement in the 2-week post-operative period. The patient denied other symptoms, including bloating, diarrhea, hematochezia, or urinary symptoms. The patient initially reported to an outside emergency room on postoperative day 10, with these same complaints. At that time, she was discharged with famotidine and pain control and encouraged to follow up with general surgery. Three months prior to her gastrectomy, she had been admitted to the hospital for pancreatitis, managed with fluids and analgesics, and discharged after two days, with resolution of her symptoms.

**Figure 1:**
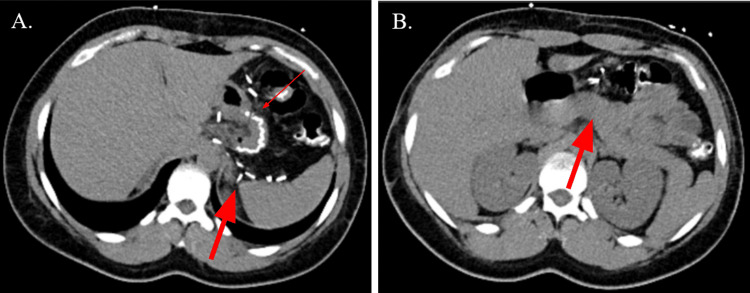
Non-contrast CT abdomen and pelvis (A) Axial view of the abdomen demonstrating several clips for vessel sealing (thick arrow) and the staple line (thin arrow). (B) Axial view of the abdomen at the level of the pancreas (thick arrow) without signs of acute inflammation.

**Figure 2:**
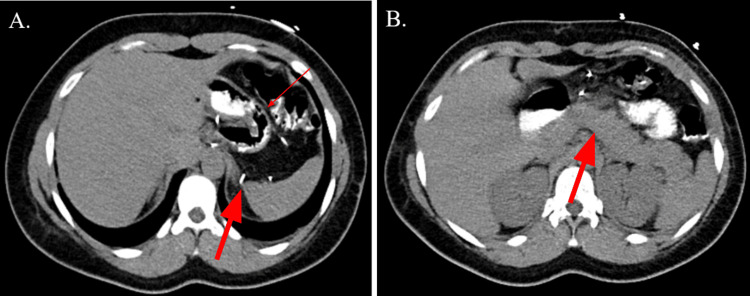
CT abdomen and pelvis with oral contrast (axial view) (A) Axial view of the abdomen demonstrating several surgical clips (thick arrow) and post-surgical changes (thin arrow) associated with sleeve gastrectomy. (B) Axial view of the abdomen at the level of the pancreas (thick arrow). No bowel obstruction or signs of acute inflammation were seen.

**Figure 3:**
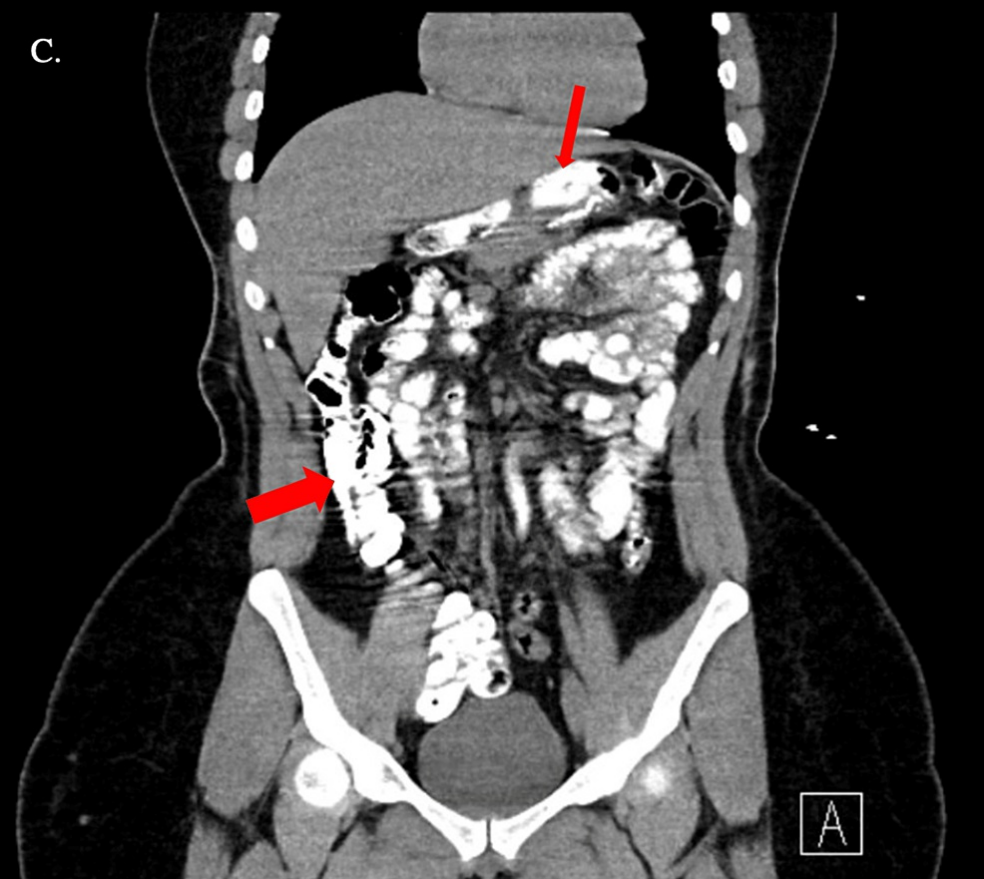
CT abdomen and pelvis with oral contrast (frontal view) Frontal view of the abdomen demonstrating post-surgical gastric sleeve with contrast in the gastric sleeve (thin arrow) and bowels (thick arrow).

Upon presentation, the patient was afebrile (98.9 °F) and hemodynamically stable (heart rate (HR) 94 bpm, respiratory rate (RR) 18 bpm, blood pressure (BP) 116/78 mmHg). BMI was 32.7 kg/m^2^ on presentation. Initial labs revealed an unremarkable comprehensive metabolic panel (CMP), complete blood count (CBC), basic metabolic panel (BMP), but an elevated lipase of 721 U/L (reference interval 73-393 U/L) and Erythrocyte Sedimentation Rate of 35 mm/hr (reference interval 0-20 mm/hr). Chest X-ray was negative for acute pathology. CT abdomen and pelvis with and without oral contrast were read as post-surgical changes associated with sleeve gastrectomy, without staple line leak. Oral contrast passed through the gastrointestinal (GI) tract freely without dilation, free air, or radiographic evidence of obstruction or perforation (Figures 1-3). The patient was administered hydromorphone, dicyclomine, metoclopramide, and ondansetron for her symptoms. She was admitted to a hospital medical-surgical unit for abdominal pain and intractable nausea. Her symptoms were initially thought to be caused by dumping syndrome due to normal radiographic appearance.

During admission, repeat labs were drawn, which showed increasing elevations in lipase (1,224 U/L, with a peak of 2,542 U/L), consistent with pancreatitis. A repeat non-contrast CT scan six days after admission showed residual contrast throughout the GI tract with no acute abnormality seen. Abdominal ultrasound was not performed due to the normal radiographic appearance of the gallbladder and biliary system, but a previous ultrasound four months prior was unremarkable.

The patient was permitted to eat a low-fat diet as tolerated and administered lactated ringers (250 cc/hr) and a multimodal pain management regimen consisting of hydromorphone and ketorolac. The general surgery service was consulted for persistent abdominal pain and recommended an esophagogastroduodenoscopy (EGD). This revealed two gastric polyps and twisting of the gastric sleeve not seen on the CT scan. The duodenum was unremarkable. Both polyps were biopsied and showed mild chronic gastritis without *H. pylori* or acute disease activity. Due to intolerance of oral intake, total parenteral nutrition (TPN) was initiated. The patient was discharged on TPN plus oral nutrition as tolerated and scheduled for outpatient follow-up with surgery for conversion to Roux-en-Y gastric bypass. The patient returned to the hospital a day after discharge, with uncontrolled abdominal pain, nausea, and vomiting. There were no significant changes in repeat labs, imaging, or endoscopy. She was treated symptomatically with intravenous (IV) fluids, ondansetron, and pain control and transferred to another medical facility 18 hours later for Roux-en-Y gastric bypass.

## Discussion

The sleeve gastrectomy is typically performed laparoscopically and involves resection of the greater curvature and fundus of the stomach. This vertical gastrectomy results in a more narrow, tubular, and less distensible stomach, with fewer ghrelin-producing cells to stimulate hunger [8].

A systematic review of sleeve gastrectomy reported a 6.2% average complication rate in non-high-risk patients and a 9.4% average complication rate in high-risk patients [8]. The most common complications include bleeding, leaks, and strictures [8,9]. Bleeding complications may arise from the anastomoses of the sleeve or the sleeve staple [9]. Most resolve spontaneously [9]. Leaks may arise at the gastroesophageal junction or the angle of His, resulting in peritonitis [8]. This complication occurs if the final staple line is placed across the gastroesophageal junction or at the distal esophagus, resulting in a staple line disruption [8]. Proximal leaks may result from a functional obstruction caused by strictures at the incisura or mid-sleeve stenosis of the sleeve [8]. Leaks can clinically manifest as unexplained tachycardia, abdominal pain, fevers, or persistent hiccups [8,9]. Upper gastrointestinal contrast studies and CT scans should be utilized to evaluate the presence of a leak [8]. Lastly, strictures may result from twisting or kinking of the sleeve and commonly occur at the incisura. Direct causes include a too narrow lumen or a sharply angulated staple line at the incisura angularis [8]. Clinical features of strictures include persistent dysphagia, nausea, and vomiting [8,9]. Treatment involves antiemetics and endoscopic balloon dilations of the obstruction [8,9].

Our patient experienced two weeks of nausea, dysphagia, and vomiting after her sleeve gastrectomy. The initial diagnosis was suspected to be dumping syndrome, as imaging showed no signs of leak or stricture formation. Dumping syndrome can be subdivided into early and late phases based on the pathophysiology and timing of symptoms. Early dumping syndrome is characterized by gastrointestinal and vasomotor symptoms resulting from rapid gastric emptying and the arrival of hyperosmolar contents in the small bowel [10]. Symptoms of abdominal pain, nausea, flushing, palpitations, diarrhea, and drowsiness occur 30 to 60 minutes after a meal [9,10]. Late dumping syndrome is characterized by the symptoms of neuroglycopenia and autonomic reactivity due to the rapid delivery of carbohydrates to the small intestines. The symptoms of fatigue, confusion, syncope, hunger, perspiration, irritability, palpitations, and tremors occur one to three hours after a meal [10]. Clinical suspicion of dumping syndrome should be raised when a patient presents with consistent symptoms and a history of recent gastric surgery. However, imaging and endoscopy should be performed first to explore other post-operative complications, including strictures [10]. Once other post-operative complications are ruled out, the diagnosis of dumping syndrome can be confirmed via the oral glucose tolerance test [10].

In our case, CT imaging was read as normal postoperative changes, but endoscopy proved to be diagnostic, revealing a twisted gastric sleeve causing a gastric outlet obstruction. The patient’s symptoms of nausea, vomiting, and abdominal pain were all secondary to the obstruction. However, the clinical picture became more complex as repeat labs revealed persistently elevated lipase levels, raising clinical suspicion of pancreatitis as a secondary diagnosis. Further review of the patient’s medical history revealed a previous diagnosis of acute pancreatitis prior to her surgery, increasing her risk of developing post-operative pancreatitis [11]. 

A cohort study reported 3.5 years as the average median time from bariatric surgery to developing acute pancreatitis, with the most common etiology being gallstones [12]. The formation of gallstones is likely facilitated by rapid weight loss after surgery [12]. Additionally, postoperative anastomotic strictures, leak, ductal dilation, sludge, and choledocholithiasis have been linked with the development of postoperative acute pancreatitis [11,12]. A multivariable analysis reported a prior history of acute pancreatitis was associated with a 7-fold increased risk in post-operative acute pancreatitis [11]. Prophylactic cholecystectomy at the time of bariatric surgery may be considered in patients with a prior history of acute pancreatitis [11]. 

Additionally, in order to prevent post-operative complications, as seen in our patient, it is crucial that twisting of the staple line or narrowing of the sleeve does not occur, as this could lead to stricture formation [8]. A bougie can be utilized to calibrate the sleeve lumen during the transection [8]. Gastropexy, surgical fixation of the stomach to the greater omentum, has also recently been studied in the prevention of all post-operative complications, including volvulus of the gastric sleeve. A meta-analysis reported a statistically significant decrease in gastric sleeve torsion and post-operative nausea in patients who underwent gastropexy during their sleeve gastrectomy procedure [13] In a case of pancreatitis secondary to a small bowel obstruction (SBO) three days after a laparoscopic Roux-en-Y gastric bypass, it was hypothesized that the SBO resulted in stasis and reflux of gastrointestinal content through the ampulla of Vater and into the pancreas, elevating lipase levels [11]. Gastropexy promotes proper sleeve orientation, which enhances gastric emptying and prevents stasis [13]. In our patient, gastropexy could have decreased her risk of developing secondary pancreatitis.

Elevated serum lipase levels can be interpreted as a sign of stasis and the beginning of pancreatitis in a post-operative bariatric patient and therefore should be given immediate attention [11]. If a patient is experiencing nausea, vomiting, and abdominal pain in the setting of elevated lipase levels, the diagnosis of acute pancreatitis should be considered [14]. If clinical symptoms are not consistent with typical pancreatitis, then a CT scan with contrast and MRI could be utilized for evaluation [14]. Early aggressive intravenous hydration should be started within the first 12 to 24 hours of symptom onset [14]. In mild pancreatitis, oral nutrition can be started as soon as tolerated, but in severe pancreatitis, enteral nutrition is recommended to avoid infectious complications [14].

## Conclusions

Sleeve gastrectomy is the most common bariatric surgery. This case presents the complication of sleeve torsion resulting in gastric outlet obstruction and secondary pancreatitis. The patient presented with postoperative nausea, vomiting, and abdominal pain. Initial imaging via CT scans with and without oral contrast did not reveal torsion or stenosis of the gastric sleeve. Endoscopy was then utilized to evaluate the integrity of the sleeve, revealing torsion of the sleeve. Lipase levels should be ordered promptly in the workup of postoperative nausea, vomiting, and abdominal pain, especially if the patient had a prior diagnosis of acute pancreatitis before surgery. As gallstones are the most common cause of postoperative acute pancreatitis, performing a prophylactic cholecystectomy during the sleeve gastrectomy may be considered to reduce the risk of postoperative acute pancreatitis. Additionally, postoperative pancreatitis can also be caused by gastric outlet obstruction. Preventative measures, including calibration of the sleeve lumen with a bougie or gastropexy, should be utilized to prevent sleeve torsion and stenosis. Management of postoperative gastric outlet obstruction includes supportive care, balloon dilation if a focus of stenosis is identified or gastric bypass if symptoms persist.
